# Understanding the mechanism of IL-1β secretion

**DOI:** 10.1016/j.cytogfr.2011.10.001

**Published:** 2011-08

**Authors:** Gloria Lopez-Castejon, David Brough

**Affiliations:** Faculty of Life Sciences, University of Manchester, AV Hill Building, Oxford Road, Manchester M13 9PT, UK

**Keywords:** IL-1β, Caspase-1, Inflammation, Golgi, Cell death

## Abstract

The cytokine interleukin-1β (IL-1β) is a key mediator of the inflammatory response. Essential for the host-response and resistance to pathogens, it also exacerbates damage during chronic disease and acute tissue injury. It is not surprising therefore that there is a huge level of interest in how this protein is produced and exported from cells. However, the mechanism of IL-1β release has proven to be elusive. It does not follow the conventional ER-Golgi route of secretion. A literature full of disparate observations arising from numerous experimental systems, has contributed to a complicated mix of diverse proposals. Here we summarise these observations and propose that secretion of IL-1β occurs on a continuum, dependent upon stimulus strength and the extracellular IL-1β requirement.

## 1 Introduction

Interleukin-1β (IL-1β) is a potent pro-inflammatory cytokine that is crucial for host-defence responses to infection and injury [1]. It is also the best characterised and most studied of the 11 IL-1 family members. It is produced and secreted by a variety of cell types although the vast majority of studies have focussed on its production within cells of the innate immune system, such as monocytes and macrophages. It is produced as an inactive 31 kDa precursor, termed pro-IL-1β, in response to molecular motifs carried by pathogens called ‘pathogen associated molecular patterns’ (PAMPs). PAMPs act through pattern recognition receptors (PRR's) on macrophages to regulate pathways that control gene expression [2]. Induction of pro-IL-1β expression is generally referred to as a priming step, and is an inefficient secretion stimulus. The primed cell must now encounter a further PAMP, or DAMP (danger associated molecular pattern, endogenous molecules released from dead cells) to induce the processing and secretion of an active IL-1β molecule.

**Fig. 1:**
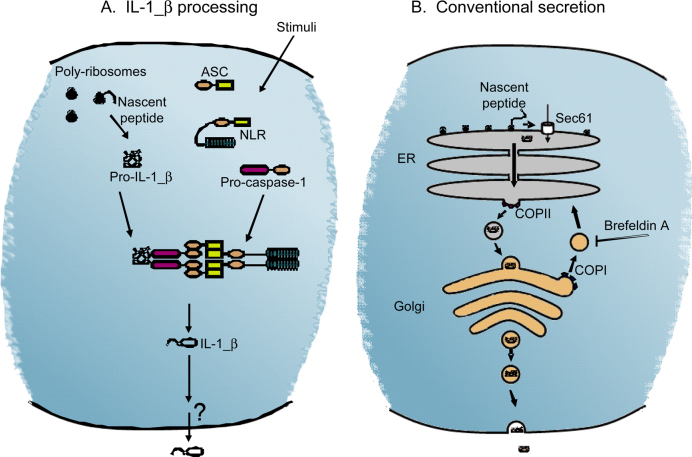
Schematic diagrams showing the components and the formation of the inflammasome, and of the conventional pathway of protein secretion. (A) Following the activation of a primed cell by an appropriate stimulus (see Section 3.1) a series of homotypic interactions take place between an adaptor molecule (ASC), a cytosolic PRR (e.g. a NLR) and pro-caspase-1 to form an inflammasome. This results in the activation of caspase-1 and the secretion of IL-1β. (B) Conventionally secreted proteins are translocated into the ER and traffic through the ER and Golgi before reaching their extracellular destination. The fungal metabolite brefeldin A inhibits the conventional pathway of protein secretion.

Pro-IL-1β is cleaved by the pro-inflammatory protease caspase-1 [3]. The activation of caspase-1 occurs *via* recruitment to a multi-protein complex termed the inflammasome (Fig. 1A); a molecular scaffold composed of adaptor molecules, a cytosolic pattern recognition receptor, and pro-caspase-1 [4]. The best characterised inflammasome, is formed by the cytosolic PRR NLRP3 [4]. NLRP3 has a tripartite structure with a PAMP/DAMP sensing C-terminal leucine rich repeat (LRR), a central nucleotide binding (or NACHT) domain and an N-terminal pyrin domain (PYD) [4]. The PYD domain of NLRP3 recruits the adaptor molecule apoptosis-associated speck-like protein containing a caspase recruitment domain (ASC) *via* a homotypic interaction with its PYD domain. Likewise, pro-caspase-1 is recruited to ASC *via* a homotypic interaction of CARD domains facilitating caspase-1 activation. Following caspase-1-dependent processing of pro-IL-1β, mature IL-1β is rapidly secreted from the cell [5]. However, *how* IL-1β is secreted from the cell is not clear. Multiple observations and proposals have been made that do not allow the formation of a single unified mechanism of secretion, but rather suggest that there are multiple mechanisms. These of course may not be mutually exclusive, but may each make a specific contribution to IL-1β-dependent inflammation. Here, we attempt to summarise these observations and to provide a rationale for the existence of the multiple mechanisms proposed.

## 2 Conventional protein secretion

The endoplasmic reticulum (ER) and Golgi apparatus together form the endo-membrane system responsible for the targeting of the vast majority of proteins to the extracellular space and to specialised sub-cellular compartments [6]. So typical is this mechanism for protein export, it has attained the status of being the ‘conventional’ pathway when compared to the secretion of a handful of proteins that utilise alternative ‘non-conventional’ routes of cellular exit [7]. Typically proteins are co-translationally translocated into the ER, although some are post-translationally translocated [8]. The first step in the co-translational pathway of translocation is the detection of a signal sequence at the N-terminus of the nascent peptide, as it emerges from the ribosome, by the signal recognition particle (SRP) [8,9]. The SRP bound to the ribosome and nascent protein chain then binds to the SRP receptor (SR) anchored on the ER membrane and subsequently the nascent peptide chain is translocated through the protein conducting channel formed by sec61α and sec61γ subunits into the ER lumen [8,9].

Once in the lumen of the ER the signal peptide is removed from the nascent protein chain which then, with the aid of chaperones, assumes a correctly folded state and accumulates at the ER exit site by virtue of its binding to the coat protein complex II apparatus (COPII) [10,11]. The COPII complex facilitates the budding of COPII-coated vesicles (that contain the cargo protein) from the ER and that subsequently fuse with the Golgi complex thereby depositing the cargo protein for the next stage of the secretory pathway [12]. The cargo proteins continue their journey through the Golgi receiving further post-translational modifications before they are packaged and transported to their final destination; another organelle, or exocytosis from the cell [13,14]. In order to maintain organelle homeostasis, and for retrieval of proteins back to the ER, Golgi membrane needs to be recycled, and this is achieved *via* the budding, retrograde transport, and fusion of COPI coated vesicles [12]. COPI-mediated retrograde transport is inhibited by the fungal metabolite brefeldin A (BFA), and treatment of cells with BFA causes the collapse of the Golgi and its redistribution into the ER, and thus completely inhibits conventional protein secretion [6,15].

That, in essence, is the route taken by conventionally secreted proteins to exit the cell (Fig. 1B). The knowledge that IL-1β lacked a signal peptide [16] led Anna Rubartelli, in 1990, to ask whether IL-1β utilised the conventional pathway of secretion by treating LPS-activated monocytes with BFA. Whilst the secretion of IL-6 and TNFα were inhibited by BFA treatment, the secretion of IL-1β was actually enhanced, thus providing the first direct evidence that IL-1β follows a non-conventional route of protein secretion [17].

## 3 IL-1 secretion

### 3.1 Secretion stimuli

When discussing the literature on the secretion of IL-1β, it is important to consider the stimulus inducing release. IL-1β is released in response to many PAMPs and DAMPs which can activate a variety, or in some cases multiple, PRR's to form inflammasomes. As discussed below, the mechanism of secretion may be influenced by stimulus type and strength. Here we summarise some of the best known and most widely used stimuli that induce IL-1β secretion. Extracellular ATP acting *via* the P2X7 receptor induces caspase-1-dependent release of IL-1β [18,19], and is dependent upon the formation of the NLRP3 inflammasome [20]. The toxins nigericin and maitotoxin, as well as infection with *Staphylococcus aureus* also induce NLRP3-dependent IL-1β release [20]. Monosodium urate (MSU) crystals, calcium pyrophosphate dihydrate (CPPD) crystals [21], cholesterol crystals [22], silica crystals and aluminium salts [23], and aggregated beta-amyloid [24] are also known activators of NLRP3. Infection of macrophages with *Salmonella typhimurium* [25], *Shigella flexneri* [26], *Legionella pneumophila* [27], and *Pseudomonas aeruginosa* [28] all induce IL-1β secretion *via* activation of an NLRC4 inflammasome. The NLRP1 inflammasome is activated by muramyl dipeptide (MDP) [29], and in mouse, NLRP1b-inflammasome is activated by *Bacillus anthracis* lethal toxin [30]. The DNA sensing receptors AIM2 [31,32] and RIG-I [33] also form inflammasomes. Infection of macrophages with *Listeria monocytogenes* induces the activation of caspase-1 *via* NLRP3, NLRC4 and AIM2 inflammasomes [34].

### 3.2 Secretion mechanisms

#### 3.2.1 Rescue and redirect

Prior to Rubartelli's observations in 1990, it was known that IL-1β is expressed as a precursor and that it lacks a signal sequence [16], and also, that it is absent from the ER and the Golgi of LPS-activated monocytes as determined by immunoelectron microscopy [35]. IL-1β is translated on free polyribosomes associated with the cytoskeleton, and not membrane-bound polyribosomes [36]. The vast majority of IL-1β in LPS-activated monocytes localises to the cytosol, although a fraction resides in vesicles and is protected from tryptic digestion [17,37]. These IL-1β-containing vesicles were subsequently identified as being of endolysosomal in nature [38]. It was proposed that a fraction of cellular IL-1β is targeted to lysosomes for degradation, but that this fraction can be rescued by triggering lysosome exocytosis and thus secretion of IL-1β. Indeed, co-incubation of LPS-stimulated monocytes with the protease inhibitors pepstatin and leupeptin enhances the release of IL-1β supporting this hypothesis [38]. How does cytosolic IL-1β get sequestered into vesicles? In 1990 Rubartelli suggested autophagy may provide a route [17] and recent studies suggest that this may in fact be the case [39]. Autophagy is a mechanism whereby damaged organelles or proteins in the cytosol become enclosed in a double membrane structure forming an autophagosome. These vesicles subsequently fuse with lysosomes forming autolysosomes resulting in the proteolytic degradation of their contents [40]. LPS treatment of macrophages induces the recruitment of IL-1β to autophagosomes. When autophagy is inhibited this IL-1β is secreted; when autophagy is activated the sequestered IL-1β is degraded [39]. Thus a fraction of cellular IL-1β targeted for degradation can be rescued and redirected to the extracellular environment.

#### 3.2.2 Protected release

Another route that a fraction of cellular IL-1β can take out of the cell is *via* the shedding of microvesicles from the plasma membrane [41]. This was originally observed in P2X7-receptor-stimulated LPS-treated THP-1 cells [41], and thrombin activated platelets [42]. In THP-1 cells shedding of IL-1β-containing microvesicles is preceded by flip of the lipid phosphatidyl serine (PS) to the outer leaflet of the plasma membrane [41], and in astrocytes requires the activation of acid sphingomyelinase [43]. The IL-1β contained in shed microvesicles is bioactive and may be released following contact with IL-1 receptor (IL-1RI) expressing cells [41]. IL-1β-containing microvesicles have also been isolated from P2X7-receptor-stimulated LPS-treated microglia [44], astrocytes [43], and dendritic cells [45]. Shed microvesicles from dendritic cells, containing IL-1β and caspase-1, express P2X7-receptors in their membrane. ATP-stimulation of these microvesicles induces the release of their contents and thus provides a mechanism for how this protected IL-1β can be released at target sites to elicit cellular responses [45].

IL-1β can also be secreted in a protected form by being packaged and secreted *via* exosomes [46]. Exosomes are small vesicles (50–80 nm *vs.* 100–600 nm for microvesicles [47]) that are secreted from multi-vesicular bodies (MVB's) or late endosomes. They are formed by the inward budding of the MVB membrane and contain cytosol [48]. Thus cytosolic IL-1β could be sorted into the forming exosome *via* sorting protein complexes, although this has yet to be shown. The secretion of exosomes following P2X7-receptor stimulation of LPS-treated macrophages is dependent upon ASC and NLRP3, but independent of caspase-1 [47]. What is the function of protected IL-1β? The fact that much of the IL-1β secreted from the cell appears to be directly available (see below), and that IL-1β has a very short half life in plasma [49], suggests that protected IL-1β is destined for signalling processes at sites distant to the local inflammatory lesion. Both shed microvesicles and exosomes from antigen presenting cells contain MHC II molecules and induce immunomodulatory effects [50]. Thus IL-1β could modulate immune responses induced by MHC II antigenic peptides carried by these vesicles.

#### 3.2.3 Terminal release

A consequence of caspase-1 activation in macrophages following infection by NLRC4-activating pathogenic bacteria (see Section 3.1) is a rapid, and caspase-1-dependent cell death called pyroptosis [51]. Pyroptosis is a pro-inflammatory form of cell death that causes an infected macrophage to kill itself, and at the same time release pro-inflammatory cytokines such as IL-1β and another caspase-1 substrate IL-18 [51]. The rapid caspase-1-dependent pyroptotic cell death caused by *S. typhimurium* [52], as well as the caspase-1-dependent clearance of NLRC4-activating pathogens *in vivo* [53] do not depend upon IL-1β processing, although that does occur. It seems that pyroptosis serves principally to eliminate the intracellular niche required for pathogen growth [54]. Pyroptosis of macrophages following infection with *S. typhimurium* occurs after the caspase-1-dependent formation of pores (1.1–2.4 nm in diameter) in the plasma membrane [55]. These pores cause the dissipitation of ionic gradients and the osmotic lysis of the cell. This osmotic lysis can be inhibited by the presence of glycine, yet pore formation and IL-1β release is not blocked [55]. These caspase-1-dependent pores may provide a conduit through which IL-1β passes to reach the extracellular space [51,55].

However, cell death associated with IL-1β release is not only dependent upon infection with NLRC4-activating pathogens. Over 20 years ago it was reported that stimulation of LPS-primed peritoneal macrophages with ATP or allospecific cytotoxic T-lymphocytes induces cell death in addition to IL-1β processing and release [56]. 30 min incubation with the NLRP3-activating stimulus ATP causes LPS-primed murine peritoneal macrophages to round up and bleb, which is closely followed by the release of the cytolytic marker lactate dehydrogenase (LDH) [57]. Although release of mature IL-1β precedes release of LDH [5,57,58], is does raise the suggestion that IL-1β release signals a commitment to cell death [59]. ATP-induced death of LPS-treated mouse peritoneal macrophages is caspase-1-dependent [19]. Incubation of macrophages with glycine inhibits ATP-induced cell lysis, but not the release of mature IL-1β [60], similar to the phenomenon observed following *S. typhimurium* infection described above [55]. In LPS-activated monocytes treated with the NLRP3-activating stimuli, heat killed *S. aureus*, caspase-1 and IL-1β localise to the plasma membrane, prompting the suggestion that caspase-1 may gate a membrane pore through which IL-1β transits [61]. Bone marrow derived macrophages transduced to express the IL-1 family member IL-36α, and a non-cytokine Green Fluorescence Protein (GFP), when stimulated with LPS and ATP release IL-1β, IL-36α and GFP in parallel, suggesting release is due to a change in membrane integrity [62]. Thus, there are many similarities between NLRC4-mediated pyroptosis and NLRP3-mediated cell death following P2X7-receptor stimulation. The mechanisms of release described in the previous sections however are reported to be non-cytolytic (see Sections 3.2.1 and 3.2.2) even though the use of ATP as a secretion stimulus is common. The possible reasons for the differences in IL-1β release and commitment to cell death are discussed in detail below.

Cell lysis *per se* does not automatically induce release of processed IL-1β. LPS-stimulated murine peritoneal macrophages release exclusively pro-IL-1β when injured by scraping, excessive heat, freeze thaw or oxidative injury by H_2_O_2_ treatment [56]. Likewise, when treated with the Ca^2+^ ionophore A23187, or the detergent saponin, unprocessed pro-IL-1β is the only form of IL-1 found extracellularly [58]. Although not biologically active, the release of pro-IL-1β following necrotic cell death may not be without relevance. In models of sterile tissue injury [63] and acute arthritis [64,65], a caspase-1-independent activation of IL-1β is reported. A number of neutrophil derived proteases are known to cleave pro-IL-1β into biologically active molecules [63,66], with a demonstrated role for proteinase 3 in acute arthritis [64]. Thus, released pro-IL-1β following tissue injury and cell necrosis could be cleaved extracellularly by proteases derived from infiltrating neutrophils resulting in an IL-1β-dependent inflammatory response independent of caspase-1. Pro-IL-1β can also be processed into biologically active molecules by proteases from *S. aureus* [67] and *Candida albicans* [68] suggesting that extracellular processing may also occur at inflammatory lesions caused by infection.

## 4 Spectrum of secretion

**Fig. 2:**
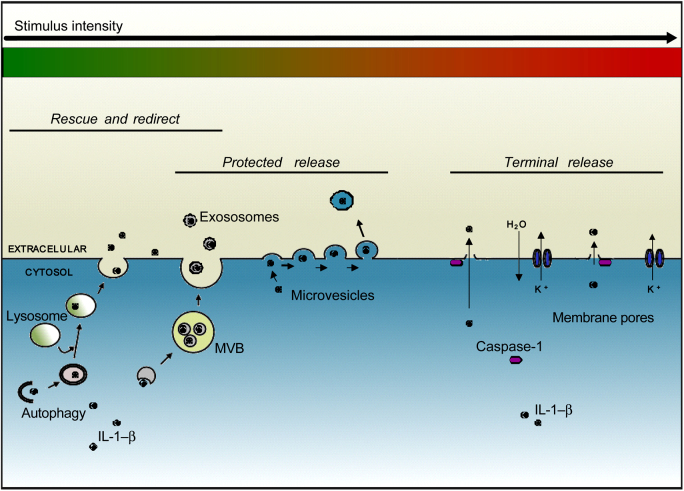
The continuum of IL-1β secretion. The secretion of IL-1β is reported to occur *via* a number of mechanisms. This figure illustrates these mechanisms as part of a continuum. The mechanism recruited may be dependent upon the strength of the inflammatory stimulus as perceived by the cell. The secretion mechanisms are classified as *Rescue and redirect*, *protected release* and *terminal release*.

Cell type, species, source and concentration of PAMPs and DAMPs, not to mention their nature and the duration of the stimulation are all factors that introduce variation and that may account for the predominance of one mechanism of secretion over another in many of the studies discussed above. Other important factors are likely to include the cells microenvironment, where temperature and pH [69], redox balance [70,71], osmolarity [72], culture conditions and time in culture [73] influence IL-1β release, as does the polarisation state of the macrophage (i.e. M1-M2) [74]. Thus the strength of the inflammatory input as perceived by the cell may vary widely from one study to another. It may thus be possible to view the different observations on the mechanisms of IL-1β secretion as belonging to a spectrum, or continuum, of release (Fig. 2). Depending upon the influencing factors described above, a cell may be induced to release a low level of IL-1β without a commitment to cell death. As the inflammatory insult escalates more mechanisms become engaged or employed to the extent that the cell can no longer maintain the latency of the plasma membrane and a commitment to cell death results. For example, microvesicle shedding is suggested as an early and rapid mechanism for the release of IL-1β, although it is followed by a slower, non-protected release of IL-1β from the cell [41]. Following ATP-induced P2X7 receptor activation on macrophages and dendritic cells both exosomes and shed microvesicles are detected in the culture supernatant [47]. Lysosome exocytosis during pyroptosis does occur, although in this case is suggested as an attempt by the cell to repair caspase-1-dependent membrane pores rather than as a mechanism of cytokine secretion [75]. These studies suggest that multiple mechanisms may be engaged for the release of IL-1β within the same cell population.

## 5 Inhibiting secretion

Although essential for resistance to infections, IL-1β also exacerbates damage during chronic disease and acute tissue injuries [76]. Thus the release mechanisms of IL-1β represent a therapeutic target. ATP-induced IL-1β release is abolished in macrophages isolated from caspase-1 KO mice [77]. Given that processing and secretion are thus so closely coupled, perhaps the most effective way of inhibiting IL-1β release is by inhibiting caspase-1. Peptide inhibitors corresponding to the cleavage site on IL-1 (YVAD) [3] are effective and widely used inhibitors of caspase-1, and block release of IL-1β [78]. Indirect inhibitors of caspase-1 are also effective. The sulfonyl urea drug glyburide, and related sulfonyl ureas inhibit ATP-induced IL-1β release [79] by inhibiting the NLRP3 inflammasome [80]. Glyburide also inhibits the accumulation of IL-1β into secretory lysosomes [38]. Lysosomal membrane rupture and cathepsin B activity are important for the activation of the NLRP3 inflammasome in response to some stimuli, where inhibition of cathepsin B attenuates IL-1β release [23,24]. In the past many diverse pharmacological effectors such as the phospholipase inhibitor bromoenol lactone [81,82], the protein phosphatase inhibitor okadaic acid and hypertonic buffer [72], anion transport inhibitors [83], alkylating agents [59], redox active drugs [70], histone deacetylase inhibitors [84], acid sphingomyelinase inhibitors [43], have been reported to inhibit IL-1β release. Given that the mechanisms of action and targets of many of these drugs remain unclear there is great potential for further discovery and exploitation of this pathway.

## 6 Conclusions and future perspectives

IL-1β is a potent pro-inflammatory cytokine produced by cells of the innate immune system. It is produced without a signal sequence and does not follow the conventional route of protein secretion, but rather employs one or more non-conventional pathways of secretion. In an attempt to consolidate the many disparate observations on IL-1β release from the literature, and to provide a rationale for their existence, we have suggested that all mechanisms are part of one continuum of secretion, or a spectrum (Fig. 2), where the routes of secretion employed are dictated by the strength of the inflammatory stimulus and thus the levels of IL-1β required extracellularly to mount an effective inflammatory response. We propose that there are, in general, three categories of secretion mechanism. For their classification in this review we have labelled them as *Rescue and redirect*, *protected release* and *terminal release*. We discuss evidence to suggest that more than one of these mechanisms can be engaged within the same cell population at any one time adding credence to our proposal.

*Rescue and redirect* is based on Rubartelli's observation that there is a fraction of cellular IL-1β localised to vesicles of endolysosomal nature that is targeted for degradation, but that can be redirected to the extracellular space following an appropriate secretion stimulus [38]. The regulated secretion of lysosomes is crucial for cells of hemopoietic linage and can occur in response to a variety of stimuli [85]. It is non-lytic and given that only a small fraction of cellular IL-1β is localised to vesicles [17], such a mechanism will likely be engaged when the extracellular requirement for IL-1β is low, or perhaps to supplement extracellular IL-1β provided by other mechanisms. *Protected release* is based upon, firstly the observation that bioactive IL-1β can be found in shed microvesicles from the plasma membrane [41], and subsequently that it is also found in secreted exosomes [46]. Since IL-1β has a very short half life in plasma, it makes sense to suggest that protected IL-1β is destined for sites distant to the inflammatory lesion. In ATP stimulated THP-1 cells there is, in addition to microvesicle contained IL-1β, vesicle free IL-1β released into the supernatant [41]. Thus, protected release occurs alongside additional mechanisms providing locally available IL-1β. *Terminal release* involves a commitment to cell death and occurs, we propose, under conditions of extreme inflammatory stress. Such a mechanism seems geared for the rapid release of large quantities of active IL-1β directly across a disintegrating plasma membrane. It may be possible not to exceed a threshold of plasma membrane latency before the commitment to cell death becomes irreversible.

Due to the diversity of secretion stimuli, culture conditions, the cell types that secrete IL-1β and the difficulty of their genetic manipulation, elucidating the precise mechanisms of IL-1β secretion remains a considerable challenge. By viewing the non-conventional secretion of IL-1β as a continuum dependent upon the extracellular requirement of IL-1β, it has been possible to rationalise a context in which all mechanisms contribute to the secretion of IL-1β. Given that there is still so much biology to be discovered about IL-1β and that there is a collective curiosity within the field about how its released, the picture of IL-1β release mechanisms is likely to be dynamic with many future insights yet to come.

## References

[1] Dinarello C.A. (1996). Biologic basis for interleukin-1 in disease. Blood.

[2] Takeuchi O., Akira S. (2010). Pattern recognition receptors and inflammation. Cell.

[3] Thornberry N.A., Bull H.G., Calaycay J.R., Chapman K.T., Howard A.D., Kostura M.J. (1992). A novel heterodimeric cysteine protease is required for interleukin-1 beta processing in monocytes. Nature.

[4] Schroder K., Tschopp J. (2010). The inflammasomes. Cell.

[5] Brough D., Rothwell N.J. (2007). Caspase-1-dependent processing of pro-interleukin-1 beta is cytosolic and precedes cell death. J Cell Sci.

[6] Rothman J.E. (1994). Mechanisms of intracellular protein transport. Nature.

[7] Nickel W., Rabouille C. (2009). Mechanisms of regulated unconventional protein secretion. Nat Rev Mol Cell Biol.

[8] Cross B.C., Sinning I., Luirink J., High S. (2009). Delivering proteins for export from the cytosol. Nat Rev Mol Cell Biol.

[9] Schwartz T.U. (2007). Origins and evolution of cotranslational transport to the ER. Adv Exp Med Biol.

[10] Dancourt J., Barlowe C. (2010). Protein sorting receptors in the early secretory pathway. Annu Rev Biochem.

[11] Anelli T., Sitia R. (2008). Protein quality control in the early secretory pathway. EMBO J.

[12] Bonifacino J.S., Glick B.S. (2004). The mechanisms of vesicle budding and fusion. Cell.

[13] Jackson C.L. (2009). Mechanisms of transport through the Golgi complex. J Cell Sci.

[14] Emr S., Glick B.S., Linstedt A.D., Lippincott-Schwartz J., Luini A., Malhotra V. (2009). Journeys through the Golgi – taking stock in a new era. J Cell Biol.

[15] Chardin P., McCormick F. (1999). Brefeldin A: the advantage of being uncompetitive. Cell.

[16] Auron P.E., Webb A.C., Rosenwasser L.J., Mucci S.F., Rich A., Wolff S.M. (1984). Nucleotide sequence of human monocyte interleukin 1 precursor cDNA. Proc Natl Acad Sci U S A.

[17] Rubartelli A., Cozzolino F., Talio M., Sitia R. (1990). A novel secretory pathway for interleukin-1 beta, a protein lacking a signal sequence. EMBO J.

[18] Solle M., Labasi J., Perregaux D.G., Stam E., Petrushova N., Koller B.H. (2001). Altered cytokine production in mice lacking P2X(7) receptors. J Biol Chem.

[19] Le Feuvre R.A., Brough D., Iwakura Y., Takeda K., Rothwell N.J. (2002). Priming of macrophages with lipopolysaccharide potentiates P2X7-mediated cell death via a caspase-1-dependent mechanism, independently of cytokine production. J Biol Chem.

[20] Mariathasan S., Weiss D.S., Newton K., McBride J., O‘Rourke K., Roose-Girma M. (2006). Cryopyrin activates the inflammasome in response to toxins and ATP. Nature.

[21] Martinon F., Petrilli V., Mayor A., Tardivel A., Tschopp J. (2006). Gout-associated uric acid crystals activate the NALP3 inflammasome. Nature.

[22] Duewell P., Kono H., Rayner K.J., Sirois C.M., Vladimer G., Bauernfeind F.G. (2010). NLRP3 inflammasomes are required for atherogenesis and activated by cholesterol crystals. Nature.

[23] Hornung V., Bauernfeind F., Halle A., Samstad E.O., Kono H., Rock K.L. (2008). Silica crystals and aluminum salts activate the NALP3 inflammasome through phagosomal destabilization. Nat Immunol.

[24] Halle A., Hornung V., Petzold G.C., Stewart C.R., Monks B.G., Reinheckel T. (2008). The NALP3 inflammasome is involved in the innate immune response to amyloid-beta. Nat Immunol.

[25] Mariathasan S., Newton K., Monack D.M., Vucic D., French D.M., Lee W.P. (2004). Differential activation of the inflammasome by caspase-1 adaptors ASC and Ipaf. Nature.

[26] Suzuki T., Franchi L., Toma C., Ashida H., Ogawa M., Yoshikawa Y. (2007). Differential regulation of caspase-1 activation, pyroptosis, and autophagy via Ipaf and ASC in Shigella-infected macrophages. PLoS Pathogens.

[27] Amer A., Franchi L., Kanneganti T.D., Body-Malapel M., Ozoren N., Brady G. (2006). Regulation of Legionella phagosome maturation and infection through flagellin and host Ipaf. J Biol Chem.

[28] Franchi L., Stoolman J., Kanneganti T.D., Verma A., Ramphal R., Nunez G. (2007). Critical role for Ipaf in *Pseudomonas aeruginosa*-induced caspase-1 activation. Eur J Immunol.

[29] Faustin B., Lartigue L., Bruey J.M., Luciano F., Sergienko E., Bailly-Maitre B. (2007). Reconstituted NALP1 inflammasome reveals two-step mechanism of caspase-1 activation. Mol Cell.

[30] Boyden E.D., Dietrich W.F. (2006). Nalp1b controls mouse macrophage susceptibility to anthrax lethal toxin. Nat Genet.

[31] Burckstummer T., Baumann C., Bluml S., Dixit E., Durnberger G., Jahn H. (2009). An orthogonal proteomic–genomic screen identifies AIM2 as a cytoplasmic DNA sensor for the inflammasome. Nat Immunol.

[32] Fernandes-Alnemri T., Yu J.W., Datta P., Wu J., Alnemri E.S. (2009). AIM2 activates the inflammasome and cell death in response to cytoplasmic DNA. Nature.

[33] Poeck H., Bscheider M., Gross O., Finger K., Roth S., Rebsamen M. (2010). Recognition of RNA virus by RIG-I results in activation of CARD9 and inflammasome signaling for interleukin 1 beta production. Nat Immunol.

[34] Wu J., Fernandes-Alnemri T., Alnemri E.S. (2010). Involvement of the AIM2, NLRC4, and NLRP3 inflammasomes in caspase-1 activation by *Listeria monocytogenes*. J Clin Immunol.

[35] Singer I.I., Scott S., Hall G.L., Limjuco G., Chin J., Schmidt J.A. (1988). Interleukin 1 beta is localized in the cytoplasmic ground substance but is largely absent from the Golgi apparatus and plasma membranes of stimulated human monocytes. J Exp Med.

[36] Stevenson F.T., Torrano F., Locksley R.M., Lovett D.H. (1992). Interleukin 1: the patterns of translation and intracellular distribution support alternative secretory mechanisms. J Cell Physiol.

[37] Matsushima K., Taguchi M., Kovacs E.J., Young H.A., Oppenheim J.J. (1986). Intracellular localization of human monocyte associated interleukin 1 (IL 1) activity and release of biologically active IL 1 from monocytes by trypsin and plasmin. J Immunol.

[38] Andrei C., Dazzi C., Lotti L., Torrisi M.R., Chimini G., Rubartelli A. (1999). The secretory route of the leaderless protein interleukin 1beta involves exocytosis of endolysosome-related vesicles. Mol Biol Cell.

[39] Harris J., Hartman M., Roche C., Zeng S.G., O‘Shea A., Sharp F.A. (2011). Autophagy controls IL-1beta secretion by targeting pro-IL-1beta for degradation. J Biol Chem.

[40] Moreau K., Luo S., Rubinsztein D.C. (2010). Cytoprotective roles for autophagy. Curr Opin Cell Biol.

[41] MacKenzie A., Wilson H.L., Kiss-Toth E., Dower S.K., North R.A., Surprenant A. (2001). Rapid secretion of interleukin-1beta by microvesicle shedding. Immunity.

[42] Lindemann S., Tolley N.D., Dixon D.A., McIntyre T.M., Prescott S.M., Zimmerman G.A. (2001). Activated platelets mediate inflammatory signaling by regulated interleukin 1beta synthesis. J Cell Biol.

[43] Bianco F., Perrotta C., Novellino L., Francolini M., Riganti L., Menna E. (2009). Acid sphingomyelinase activity triggers microparticle release from glial cells. EMBO J.

[44] Bianco F., Pravettoni E., Colombo A., Schenk U., Moller T., Matteoli M. (2005). Astrocyte-derived ATP induces vesicle shedding and IL-1 beta release from microglia. J Immunol.

[45] Pizzirani C., Ferrari D., Chiozzi P., Adinolfi E., Sandona D., Savaglio E. (2007). Stimulation of P2 receptors causes release of IL-1beta-loaded microvesicles from human dendritic cells. Blood.

[46] Qu Y., Franchi L., Nunez G., Dubyak G.R. (2007). Nonclassical IL-1 beta secretion stimulated by P2X7 receptors is dependent on inflammasome activation and correlated with exosome release in murine macrophages. J Immunol.

[47] Qu Y., Ramachandra L., Mohr S., Franchi L., Harding C.V., Nunez G. (2009). P2X7 receptor-stimulated secretion of MHC class II-containing exosomes requires the ASC/NLRP3 inflammasome but is independent of caspase-1. J Immunol.

[48] Record M., Subra C., Silvente-Poirot S., Poirot M. (2011). Exosomes as intercellular signalosomes and pharmacological effectors. Biochem Pharmacol.

[49] Kudo S., Mizuno K., Hirai Y., Shimizu T. (1990). Clearance and tissue distribution of recombinant human interleukin 1 beta in rats. Cancer Res.

[50] Thery C., Ostrowski M., Segura E. (2009). Membrane vesicles as conveyors of immune responses. Nat Rev Immunol.

[51] Bergsbaken T., Fink S.L., Cookson B.T. (2009). Pyroptosis: host cell death and inflammation. Nat Rev Microbiol.

[52] Monack D.M., Detweiler C.S., Falkow S. (2001). Salmonella pathogenicity island 2-dependent macrophage death is mediated in part by the host cysteine protease caspase-1. Cell Microbiol.

[53] Miao E.A., Leaf I.A., Treuting P.M., Mao D.P., Dors M., Sarkar A. (2010). Caspase-1-induced pyroptosis is an innate immune effector mechanism against intracellular bacteria. Nat Immunol.

[54] Brodsky I.E., Medzhitov R. (2011). Pyroptosis: macrophage suicide exposes hidden invaders. Curr Biol.

[55] Fink S.L., Cookson B.T. (2006). Caspase-1-dependent pore formation during pyroptosis leads to osmotic lysis of infected host macrophages. Cell Microbiol.

[56] Hogquist K.A., Nett M.A., Unanue E.R., Chaplin D.D. (1991). Interleukin 1 is processed and released during apoptosis. Proc Nat Acad Sci U S A.

[57] Perregaux D., Gabel C.A. (1994). Interleukin-1 beta maturation and release in response to ATP and nigericin. Evidence that potassium depletion mediated by these agents is a necessary and common feature of their activity. J Biol Chem.

[58] Perregaux D., Barberia J., Lanzetti A.J., Geoghegan K.F., Carty T.J., Gabel C.A. (1992). IL-1 beta maturation: evidence that mature cytokine formation can be induced specifically by nigericin. J Immunol.

[59] Laliberte R.E., Eggler J., Gabel C.A. (1999). ATP treatment of human monocytes promotes caspase-1 maturation and externalization. J Biol Chem.

[60] Verhoef P.A., Kertesy S.B., Lundberg K., Kahlenberg J.M., Dubyak G.R. (2005). Inhibitory effects of chloride on the activation of caspase-1, IL-1beta secretion, and cytolysis by the P2X7 receptor. J Immunol.

[61] Singer I.I., Scott S., Chin J., Bayne E.K., Limjuco G., Weidner J. (1995). The interleukin-1 beta-converting enzyme (ICE) is localized on the external cell surface membranes and in the cytoplasmic ground substance of human monocytes by immuno-electron microscopy. J Exp Med.

[62] Martin U., Scholler J., Gurgel J., Renshaw B., Sims J.E., Gabel C.A. (2009). Externalization of the leaderless cytokine IL-1F6 occurs in response to lipopolysaccharide/ATP activation of transduced bone marrow macrophages. J Immunol.

[63] Fantuzzi G., Ku G., Harding M.W., Livingston D.J., Sipe J.D., Kuida K. (1997). Response to local inflammation of IL-1 beta-converting enzyme-deficient mice. J Immunol.

[64] Joosten L.A., Netea M.G., Fantuzzi G., Koenders M.I., Helsen M.M., Sparrer H. (2009). Inflammatory arthritis in caspase 1 gene-deficient mice: contribution of proteinase 3 to caspase 1-independent production of bioactive interleukin-1beta. Arthritis Rheum.

[65] Guma M., Ronacher L., Liu-Bryan R., Takai S., Karin M., Corr M. (2009). Caspase 1-independent activation of interleukin-1beta in neutrophil-predominant inflammation. Arthritis Rheum.

[66] Stehlik C. (2009). Multiple interleukin-1beta-converting enzymes contribute to inflammatory arthritis. Arthritis Rheum.

[67] Black R.A., Kronheim S.R., Cantrell M., Deeley M.C., March C.J., Prickett K.S. (1988). Generation of biologically active interleukin-1 beta by proteolytic cleavage of the inactive precursor. J Biol Chem.

[68] Beausejour A., Grenier D., Goulet J.P., Deslauriers N. (1998). Proteolytic activation of the interleukin-1beta precursor by *Candida albicans*. Infect Immun.

[69] Rubartelli A., Bajetto A., Allavena G., Cozzolino F., Sitia R. (1993). Post-translational regulation of interleukin 1 beta secretion. Cytokine.

[70] Tassi S., Carta S., Delfino L., Caorsi R., Martini A., Gattorno M. (2010). Altered redox state of monocytes from cryopyrin-associated periodic syndromes causes accelerated IL-1beta secretion. Proc Natl Acad Sci U S A.

[71] Carta S., Tassi S., Pettinati I., Delfino L., Dinarello C.A., Rubartelli A. (2011). The rate of interleukin-1{beta} secretion in different myeloid cells varies with the extent of redox response to toll-like receptor triggering. J Biol Chem.

[72] Perregaux D.G., Laliberte R.E., Gabel C.A. (1996). Human monocyte interleukin-1beta posttranslational processing. Evidence of a volume-regulated response. J Biol Chem.

[73] Laliberte R.E., Perregaux D.G., McNiff P., Gabel C.A. (1997). Human monocyte ATP-induced IL-1 beta posttranslational processing is a dynamic process dependent on in vitro growth conditions. J Leukocyte Biol.

[74] Pelegrin P., Surprenant A. (2009). Dynamics of macrophage polarization reveal new mechanism to inhibit IL-1beta release through pyrophosphates. EMBO J.

[75] Bergsbaken T., Fink S.L., den Hartigh A.B., Loomis W.P., Cookson B.T. (2011). Coordinated host responses during pyroptosis: caspase-1-dependent lysosome exocytosis and inflammatory cytokine maturation. J Immunol.

[76] Dinarello C.A. (2010). Anti-inflammatory agents: present and future. Cell.

[77] Brough D., Le Feuvre R.A., Wheeler R.D., Solovyova N., Hilfiker S., Rothwell N.J. (2003). Ca^2+^ stores and Ca^2+^ entry differentially contribute to the release of IL-1 beta and IL-1 alpha from murine macrophages. J Immunol.

[78] Sanz J.M., Di Virgilio F. (2000). Kinetics and mechanism of ATP-dependent IL-1 beta release from microglial cells. J Immunol.

[79] Perregaux D.G., McNiff P., Laliberte R., Hawryluk N., Peurano H., Stam E. (2001). Identification and characterization of a novel class of interleukin-1 post-translational processing inhibitors. J Pharmacol Exp Ther.

[80] Lamkanfi M., Mueller J.L., Vitari A.C., Misaghi S., Fedorova A., Deshayes K. (2009). Glyburide inhibits the cryopyrin/Nalp3 inflammasome. J Cell Biol.

[81] Andrei C., Margiocco P., Poggi A., Lotti L.V., Torrisi M.R., Rubartelli A. (2004). Phospholipases C and A2 control lysosome-mediated IL-1 beta secretion: implications for inflammatory processes. Proc Natl Acad Sci U S A.

[82] Franchi L., Chen G., Marina-Garcia N., Abe A., Qu Y., Bao S. (2009). Calcium-independent phospholipase A2 beta is dispensable in inflammasome activation and its inhibition by bromoenol lactone. J Innate Immun.

[83] Perregaux D.G., Svensson L., Gabel C.A. (1996). Tenidap and other anion transport inhibitors disrupt cytolytic T lymphocyte-mediated IL-1 beta post-translational processing. J Immunol.

[84] Carta S., Tassi S., Semino C., Fossati G., Mascagni P., Dinarello C.A. (2006). Histone deacetylase inhibitors prevent exocytosis of interleukin-1beta-containing secretory lysosomes: role of microtubules. Blood.

[85] Stinchcombe J.C., Griffiths G.M. (1999). Regulated secretion from hemopoietic cells. J Cell Biol.

